# Copper Oxide Microtufts on Natural Fractals for Efficient Water Harvesting

**DOI:** 10.1021/acs.langmuir.0c03497

**Published:** 2021-03-11

**Authors:** Vipul Sharma, Harri Ali-Löytty, Anastasia Koivikko, Kyriacos Yiannacou, Kimmo Lahtonen, Veikko Sariola

**Affiliations:** † Faculty of Medicine and Health Technology, Tampere University, Korkeakoulunkatu 3, 33720 Tampere, Finland; ‡ Surface Science Group, Photonics Laboratory, Tampere University, P.O. Box 692, FI-33014 Tampere, Finland; § Faculty of Engineering and Natural Sciences, Tampere University, P.O. Box 692, 33014 Tampere, Finland

## Abstract

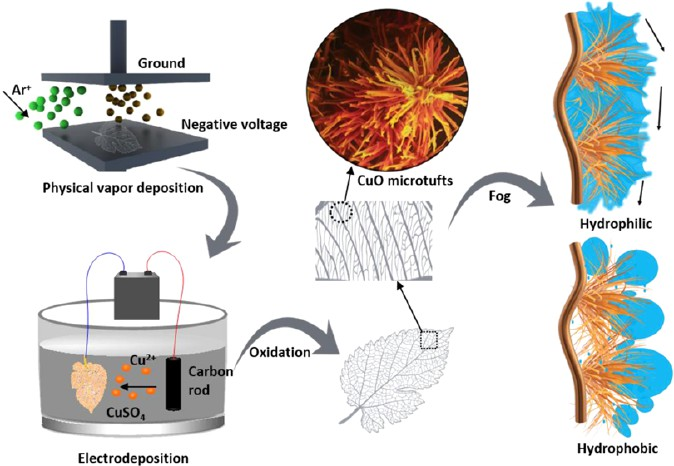

Hierarchical surfaces that aid in the droplet nucleation, growth, and removal is highly desirable for fog and moisture harvesting applications. Taking inspiration from the unique architecture of leaf skeletons, we present a multiscale surface capable of rapidly nucleating, growing, and directional transport of the water droplets. Copper oxide microtufts were fabricated onto the *Ficus religiosa* leaf skeletons via electroplating and chemical oxidation techniques. The fabricated surfaces with microtufts had high wettability and very good fog harvesting ability. CuO surfaces tend to become hydrophobic over time because of the adsorption of the airborne species. The surfaces were efficient in fog harvesting even when the hydrophobic coating is present. The overall water collection efficiencies were determined, and the role of the microtufts, fractal structures, and the orientation of leaf veins was investigated. Compared to the planar control surfaces, the noncoated and hydrophobic layer-coated copper oxide microtufts on the leaf skeletons displayed a significant increase in the fog harvesting efficiency. For superhydrophilic skeleton surfaces, the water collection rate was also observed to slightly vary with the vein orientation. The CuO microtufts along with high surface area fractals allowed an effective and sustainable way to capture and transport water. The study is expected to provide valuable insights into the design and fabrication of sustainable and efficient fog harvesting systems.

## Introduction

Billions of people around the world are suffering from the lack of drinking water because of population explosion, rapid industrial development, and heavy pollution.^1^ This shortage or lack of freshwater resources is, directly and indirectly, affecting the lives of billions of people across the globe and is a matter of serious concern. In recent times, the problem has escalated beyond arid regions, and Asia,^2^ South Africa,^3^ and the Czech Republic^4^ are a few examples. Desalination is one of the important methods currently to resolve the water crisis and mainly involves distillation-based mechanisms.^5^ However, desalination processes require a tremendous amount of energy and high operating costs, meaning huge investments, high maintenance costs, and significant carbon emission.^6^ Apart from the desalination of seawater, water collection from the atmosphere (moisture and fog) is a promising method to address the water crisis.^7−9^ This method is relatively simple, cost-effective, and is a sustainable way for water collection.

Numerous flora and fauna species can collect water from fog, and their study may offer endless inspirations for scientific research.^10−14^ Some grass such as *Cynodon dactylon*(12) and *Stipagrostis sabulicolahas*(15) have unique conical microstructures that help in the collection of fog from the air. *Stenocara gracilipes*, a type of Namib desert beetle, can efficiently capture water from humid air using the unique hydrophilic–hydrophobic structures on its back.^16^ Cactus species such as *Opuntia microdasys*(17) and other cacti belonging to arid regions^18^ rapidly collect water to fulfill their needs because of the conical spines integrated with the trichomes and other hierarchical structures. Some ferns such as *Dryopteris marginata* are known to efficiently collect the water by efficient channeling.^13^ In all of these mentioned systems, there are well-studied mechanisms related to the efficient water nucleation, growth/storage, and transport.

Based on the bioinspired principles, water harvesting materials and devices are being rapidly developed.^19−24^ Most of the bioinspired water harvesting surfaces derive inspiration from three biological systems: silk fibers from the spiders, hydrophobic/-philic patterned surfaces inspired by Namib desert beetle, and conical spines inspired by cacti. There are also many recent reports which have taken water harvesting inspiration from species such as *Nepenthes alata*,^25^*Sarracenia*,^26^ desert moss *Syntrichia caninervis*,^27^ green bristle grass,^28^ shorebirds,^29^ wheat awns,^30^*Burkheya purpureas*,^31^ etc.^20^ Arrays of hydrophilic bumps with superhydrophobic troughs on the elytra of Stenocara have been reported to accelerate the fog harvesting ability.^16^ Taking inspiration from this study, superwettable patterns involving circle array, square array, and star-shaped^32^ array were then fabricated for water collection. These surfaces had superior performances compared to conventional superhydrophilic and superhydrophobic surfaces. Fiber-like network structures inspired by the spider web and mesh were also used for water collection. Meshlike architecture is more capable than planar surfaces because it increases the Stokes’s number of fog droplets flowing around the mesh, and thus leads to increased impacts of drops with the mesh.^33^ Due to this fact, meshes are widely used in fog harvesting at many different locations.^34^ To further improve the fog collection efficiency, micro-/nanostructures were integrated onto the meshlike macroscale surfaces. The micro-/nanostructures enable the meshlike surfaces to capture fog droplets effectively by changing the microscopic wetting and nucleation properties of the surfaces. Some unique shapes such as conical microstructured arrays may also help in increasing fog capture and transport rate by the virtue of the difference in the Laplace pressure and gradient of the surface free energy. Numerous methods have been reported to fabricate the meshlike special wetting surfaces possessing micro/nanostructures.^35,36^ For example, electrospun poly(vinylidene fluoride) (PVDF) microfibers were found to improve the water collecting efficiency of commercial Raschel mesh by ∼305%.^37^ There are efforts to improve the design of meshes having multiscale hierarchical functionalities as the condensates that nucleate and grow on the surfaces tend to inhibit further water condensation.

Timely removal of the captured water is very important in any water harvesting system. The directional water transportability depends on many external factors and structural organization. These factors can delay the fog collection cycle from restarting and may lead to droplet re-evaporation. This happens by delaying the timely removal of the nucleated and grown droplet/film and even by premature removal of the nucleated droplets.^7,38^ The captured droplets usually roll or slide down the surfaces randomly and foul the nucleation sites. Therefore, it is important to look for the designs that aid in directional transport, especially in the hydrophilic surfaces. To facilitate condensate movement, straight line,^14^ wedge-shaped,^39^ and branch-patterned^40^ tracks were investigated for directional water collection. Some tracks were gravity-assisted and a few integrated Young–Laplace pressure difference and surface energy gradients to drive the condensates spontaneously and efficiently. Thus, the water collection can be increased using directional and spontaneous collection by decreasing the residual water and renewal of active surface for water collection. To improve the designs for better transport of the captured water, complex patterns combining special geometry have been reported, which includes the design inspiration from Mulberry^39^ and Banana leaves.^41^ In our previous work, we have also proposed the use of leaf-inspired superhydrophilic tracks for efficient water transport and collection.^7^ In meshlike designs also, the importance of efficient transport of water has been demonstrated and bioinspired designs have been used to facilitate water collection.

In this work, we demonstrate leaf skeleton-based three-dimensional (3D) surface for efficient water nucleation, growth, and transport. Leaf skeletons possess a fractal-like structure at the microscale along with the multiscalar interconnected veins. To increase the effective surface area and to increase droplet capture sites on the surface, copper oxide microtufts were grown onto the leaf skeletons via a combination of sputtering and electrodeposition methods. The morphological and compositional properties of copper oxide microtufts on the leaf skeleton surface are studied using field emission scanning electron microscopy (FESEM) and X-ray photoelectron spectroscopy (XPS). 3D orientation and the shape of the copper oxide microtufts provide unique wettability to the surfaces and allow them to capture tiny fog droplets from the mist stream. The interconnected veins present on the leaf skeleton help in directional transport. The veins draw the water from across the fractal network and aid in the directional and efficient collection. The surface was efficient even when it was made hydrophobic via silane treatment. The vast network of the fractals along the skeleton surface tends to facilitate the droplet growth via rapid coalescence of the droplets and eventually lead to the droplet departure. Wetting properties of the microtufts on the leaf skeleton were studied and their correlation with the fog collection capability was established. Mechanisms related to the water capture and the dynamic transport on both hydrophilic and hydrophobic leaf skeleton surfaces have been highlighted.

## Experimental Section

### Materials

For the fabrication of the leaf skeleton electrode, leaf skeletons of *Ficus religiosa* were purchased from “*Skeleton Leaf - Just the Leaves,”* United Kingdom. For electrodeposition, H_2_SO_4_ and CuSO_4_ were purchased from Merck, HCl from Romil, and poly(ethylene glycol) (PEG) was purchased from Sigma-Aldrich, Finland. For the oxidation of copper, NaOH, (NH_4_)_2_S_2_O_8_, 1*H*,1*H*,2*H*,2*H*-perfluorodecyltriethoxysilane, and CTAB were purchased from Sigma-Aldrich, Finland. All of the chemicals were used as received.

### Methods

#### Deposition of Copper Layer onto the Leaf Skeleton

To make the leaf skeleton surface suitable for the electrodeposition, a seed layer of Au (∼30 nm) was sputter-coated onto both sides of the skeleton. Cu films were electrodeposited on Au-coated leaf skeletons from an acidic plating solution including Cl^–^ promoter and PEG suppressor.^42^ Additives were chosen to promote uniform Cu electrodeposition on leaf skeleton. The plating solution consisted of 1 M H_2_SO_4_, 0.5 M CuSO_4_, 1 μM HCl, and 100 ppm PEG at 1500 g/mol (PEG 1500) and was made using CuSO_4_ (copper(II) sulfate anhydrous, 1.02791.0250 Merck Emsure), 95–97% H_2_SO_4_ (1.00731.1011 Merck Emsure), 34–37% HCl (H 396 Romil-SpA Super purity acid), PEG 1500 (86101-250G-F Sigma BioUltra), and ultrapure deionized H_2_O (18.2 MΩ cm, Merck Milli-Q).

Electrodeposition was performed in a 1 L thermostated electrochemical cell using a three-electrode system controlled by an Autolab PGSTAT204 potentiostat (Metrohm AG, Switzerland). A graphite rod (8 mm diameter, 15 cm length, Gamry Instruments, USA) and a leakless Ag/AgCl electrode (ET069, eDAQ Pty Ltd, Australia) were used as the counter and reference electrodes, respectively. The connection to the leaf skeleton was made using a crocodile clip attached to the leaf stem. All of the electrodes were supported in the cell by a stativ.

The electrodeposition was done galvanostatically at −10 mA/cm^2^ at 30 °C under forced convection conditions induced by a magnetic stirrer. The duration of electrodeposition was fixed at 2721 s that corresponds to 10 μm/cm^2^ Cu film thickness assuming 100% current efficiency and homogeneous film thickness. For the sake of simplicity, the electrochemically active surface area was approximated as the planar projected surface area of an intact leaf. Before the cathodic electrodeposition, the sample was subjected to 10 potential cycles between −0.35 and +0.55 V at 50 mV/s for surface preconditioning. After the electrodeposition, the sample was rinsed in deionized H_2_O and blown dry with N_2_. Figure S1 shows a representative potential–time curve for the Cu electrodeposition on an Au-coated leaf skeleton. Planar control samples were prepared by electrodeposition of 10 μm Cu film on Au-coated glass sheets.

#### Chemical Oxidation of Cu to CuO

In the first step, the Cu-coated leaf skeletons and controls were cleaned with ethanol and deionized water (ELGA PURELAB Option-R7) via ultrasonication for 15 min each. The surfaces were then dried in a gentle stream of N_2_ gas. In the next step, the Cu-coated leaf skeletons and controls were gently transferred to a glass container with an aqueous solution of 1 wt % NaOH and (NH_4_)_2_S_2_O_8_ in a 1:1 ratio. The solution was stirred gently at room temperature for 15 min to make sure that the copper layer does not etch out completely. After 15 min, the sample was rinsed several times with ethyl alcohol and DI water, followed by drying with compressed N_2_. The samples were transferred to an oven and were dried at 150 °C for 2 h. This leads to the phase transition of the copper hydroxide (bluish brown) to the copper oxide (dark brown)

#### Hydrophobic Coating Procedure

To make the prepared oxide surfaces hydrophobic, fluoro silane treatment was used to decrease the surface energy. The samples (leaf skeletons with CuO microtufts and controls) were placed in a sealed desiccator with a few drops of 1*H*,1*H*,2*H*,2*H*-perfluorodecyltriethoxysilane (C_16_H_19_F_17_O_3_Si) in it. The desiccator with the samples was then kept for 2 h with a vacuum at room temperature before the samples were taken out. The silane-treated plates were then placed in an oven (∼120 °C) for 30 min to get rid of any unattached silane onto the surface.

#### Characterization Tools

The morphology of the microstructures was determined using an FESEM (field emission scanning electron microscope) (Zeiss UltraPlus) operating at 5 kV. Elemental analysis was done using an EDS (energy-dispersive X-ray spectroscopy) attachment linked to the FESEM (Oxford Instruments X-MaxN 80 EDS). The fog nucleation, growth, droplet dynamics, and water transport were studied using an optical camera (Basler acA2040-120uc, Germany).

Lens-defined selected-area X-ray photoelectron spectroscopy (SAXPS) was performed employing a DAR400 X-ray source (Mg Kα, 1253.6 eV) and an Argus hemispherical electron spectrometer (Omicron Nanotechnology GmbH). The core-level spectra were collected at a normal emission angle with a pass energy of 20 eV and in-lens aperture yielding a rectangular analysis area of 1.54 (dispersive) × 4.09 (nondispersive) mm^2^. The surface composition was identified by analyzing the main core-level transitions and Cu LMM. The background-subtracted photoelectron peaks were least-squares fitted with a combination of Gaussian–Lorentzian component line shapes. The relative atomic concentrations were calculated using Scofield photoionization cross sections and an experimentally measured transmission function of the Argus analyzer. The XPS information depths (i.e., 3 × inelastic mean free path) through C_16_H_19_F_17_O_3_Si silane overlayer using Cu 2p_3/2_ and Cu L_3_M_45_M_45_ signals were 3.6 and 7.7 nm, respectively, as calculated using the TPP2M equation.^43^

#### Fog Collection Experiments

Fog collection experiments were done by exposing surfaces to the fine mist of ultrapure water generated by two commercial cool mist ultrasonic humidifiers (Bionaire BU1300W-I). Samples were kept in a vertical orientation and facing the humidifier at 50 mm as displayed in Figure 5a. The humidifier generated a cool mist that hit the surface at the rate of 50–150 mm s^–1^ (measured using a Jessiekervin YY3 hand-split digital anemometer). This is comparable to the typical wind speed of the fog in a desert which is around 10–50 mm s^–1^.^16^ The total air flow from the humidifiers was estimated as ca. 260 ± 60 mL/h (according to the specification sheet of the manufacturer). The experimental setup had a relative humidity above 99%, and all of the measurements were conducted at room temperature. The volume of water was collected and measured with a weight balance at intervals of 10 min throughout 1 h. Three sets of trials were performed on three independent samples, and the average values along with the standard deviation were calculated. Special care was taken to ensure the same trail conditions for all measurement rounds.

## Results and Discussion

**Figure 1:**
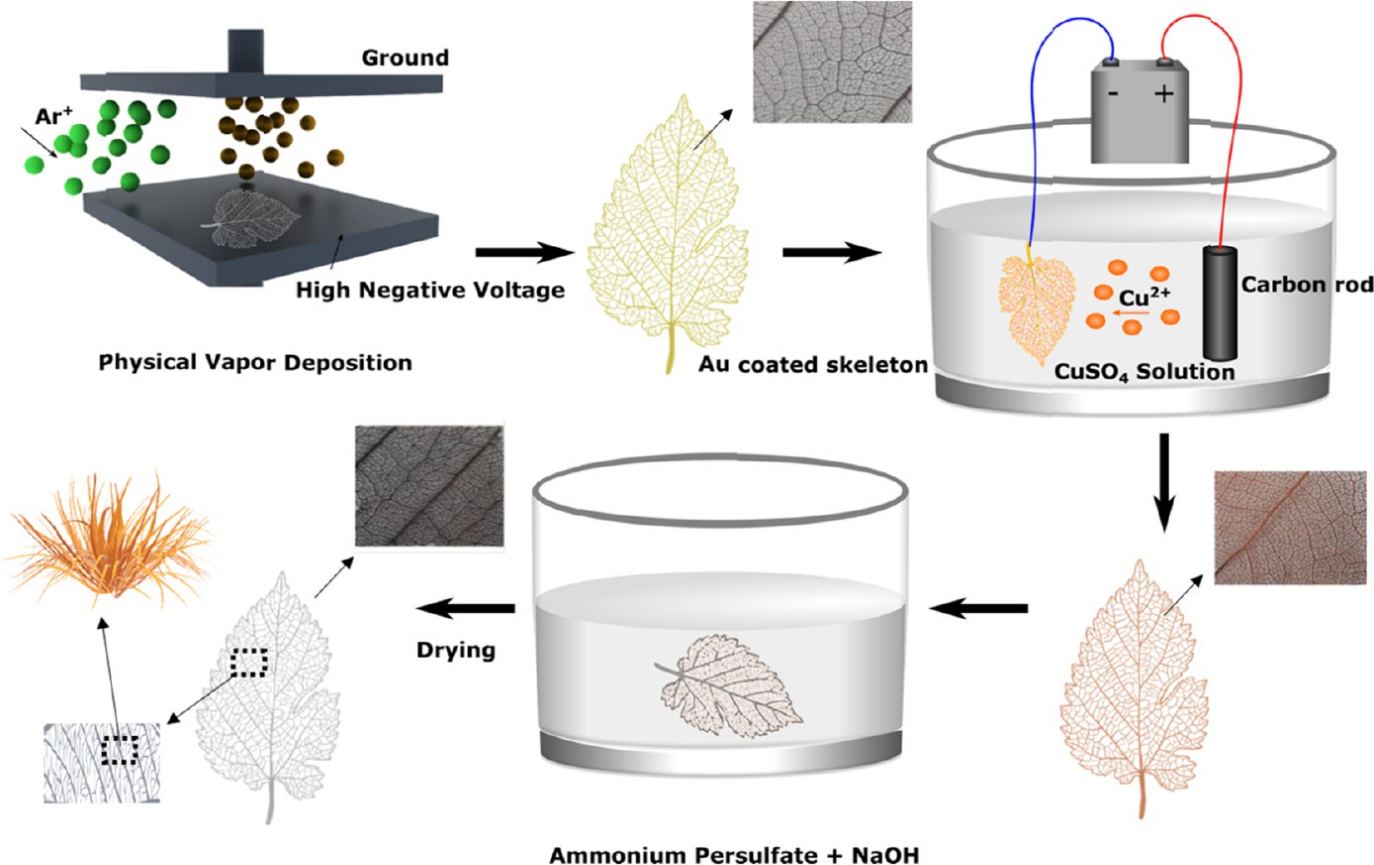
Schematics and photographs (insets) of the preparation procedure.

Figure 1 shows the fabrication of the CuO microtufts on the surface of the *F. religiosa* leaf skeleton. In this study, a combination of simple electrodeposition and oxidation method was introduced, which can coat the surface of the leaf skeleton conformally in 3D. This method is facile and can be subjected to a large scale as well.

The microtufts were conceivably composed of hydrated copper oxide that typically forms during the oxidation process of copper using NaOH and (NH_4_)_2_S_2_O_8_. The oxidation process proceeds in three steps: In the first step, Cu oxidizes rapidly to form Cu(OH)_2_ due to the presence of strong oxidizing agents NaOH and (NH_4_)2S_2_O_8_. During this process, released Cu^2+^ cations react with the hydroxide anions of the NaOH to form Cu(OH)_2_.
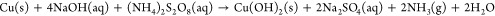
1

Gas bubbles having a characteristic ammonia odor were noted that indicated the presence of NH_3_. In the second step, Cu(OH)_2_ transforms very rapidly into tetrahydroxocuprate (II) anions Cu(OH)4^2–^ due to the alkalinity of the solution.^44^
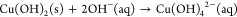
2

In the final step, a condensation reaction leads to the formation of CuO particles from Cu(OH)_4_^2–^
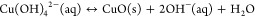
3

**Figure 2:**
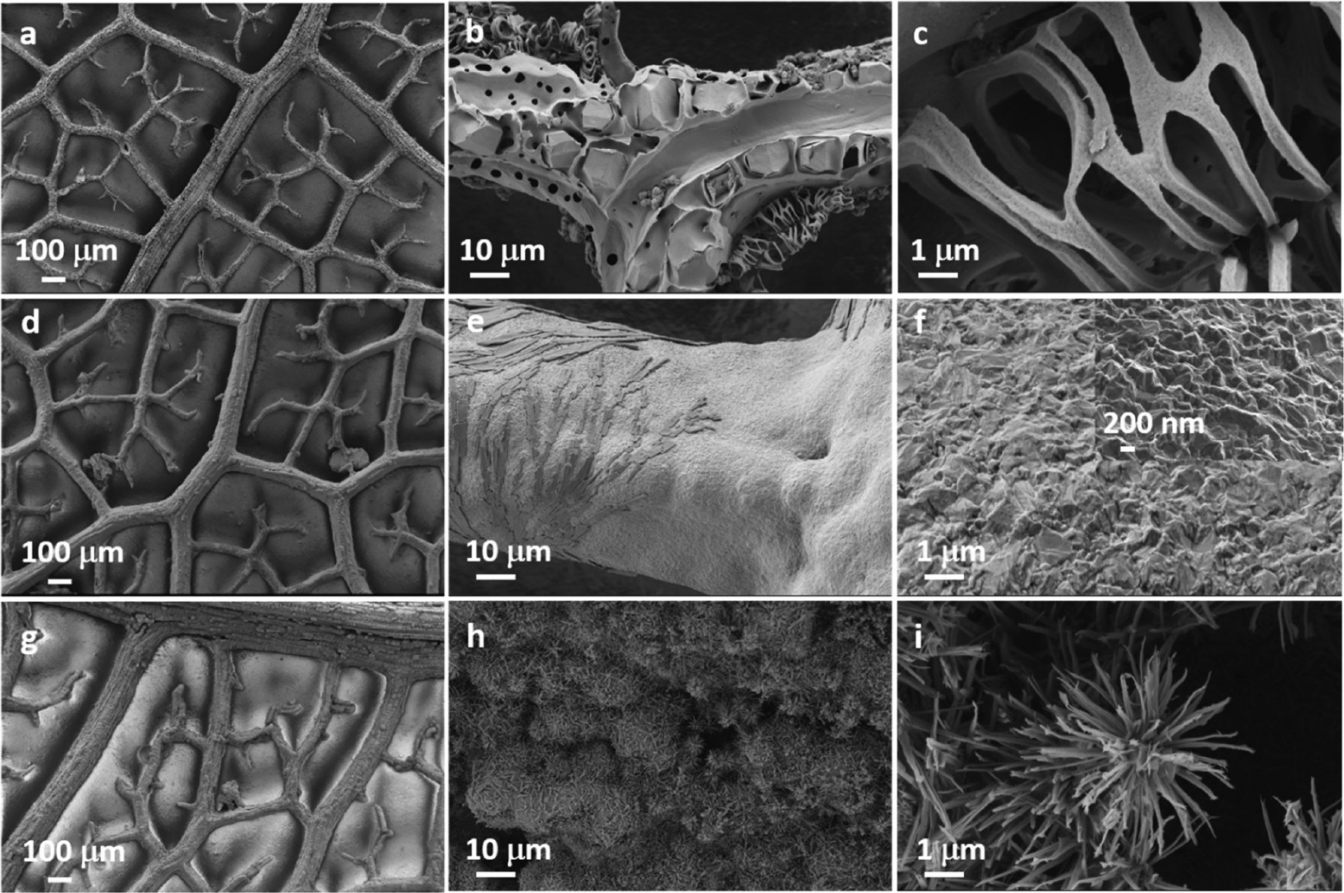
FESEM images of the leaf skeleton having Au coating (a–c), electrodeposited Cu coating (d–f), and CuO microtufts (g–i) at different resolutions.

The tiny CuO particles prepared on the surface of Cu-coated skeleton have high surface energy and are highly reactive. These small CuO particles aggregate around the nanomountains created during the Cu deposition due to the uneven surface of the leaf skeleton (Figure 2f, inset). This forms the long microfiber-like structure, and many of these fibers along with the uneven 3D geometry of the skeleton collectively make these structures look like microtufts. In the absence of these nanomountain-like structures, nanostructures in a random shape and orientation were expected. To confirm this, Cu was deposited on a planar glass substrate and was subjected to oxidation in the same way as above. As expected, the random micro-grass-like morphology was evidenced and can be seen in Figure S2 (Supporting Information). It is also noteworthy to mention that the shape and size of these copper oxide structures can be controlled by blocking of the Cu layers. For example, in our previous study, we have shown that high-density CuO nanoneedle-like morphology can be obtained by blocking the Cu layer by bisulfate ions during the oxidation process.^7^

To confirm the surface microstructures, FESEM was employed. The FESEM images at different length scales are shown in Figure 2. Figure 2a–c shows the bare leaf skeleton, where the vascular bundles along with the peripheral interconnected fibers are visible. These fibers are arranged in the fractal-like assembly and are responsible for mechanical stability and flexibility. Figure 2d–f shows the leaf skeleton after Cu deposition. It is clear from the images that the Cu has deposited conformally onto the surface as the underneath vascular bundles and fibers are not visible. Due to the irregular surface of the skeleton, the surface of the Cu displaying nano-bump or nano-mountain-like morphology is evident at the nanoscale (Figure 2f, inset). The oxidation process led to the formation of the CuO microtufts, which can be seen in Figure 2g–i. These CuO microtufts range from ∼5 to 15 μm in diameter and have high-aspect-ratio fragments. It is also evident from the micrographs that the fractal-like geometry was perfectly preserved even after the fabrication procedure and CuO microtufts are evenly distributed throughout the surface. To confirm the presence of Cu on the leaf fractal surface, energy-dispersive spectrometry (EDS) measurements were done. The EDS data corresponding to the FESEM images are shown in Figures S3–S5 (Supporting Information), which confirms the presence of Cu and CuO on the surface of the leaf skeleton. X-ray photoelectron spectroscopy analysis showed (Figure S6, Supporting Information) that before chemical oxidation treatment, the surface consisted of Cu° and some Cu^2+^ species typical for an air-oxidized Cu surface. After the chemical oxidation treatment, only Cu^2+^ species were present on the surface, confirming the chemical state of microtufts as CuO. After the application of the silane coating, the XPS spectrum was dominated by the strong F 1s signal originating from the C_16_H_19_F_17_O_3_Si silane compound and only a weak Cu LMM signal from the leaf skeleton substrate was detected. The intensity of Cu 2p signal, which is more surface sensitive than Cu LMM signal, was below the detection limit. Therefore, the average thickness of silane coating was approximated to be close to the information depth of the XPS analysis (i.e., 5–10 nm).

### Wettability

Hydrophilic surfaces are more attractive to water, and their observed wettabilities are further increased by roughness, according to the Wenzel equation^45^
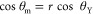
4where θ_m_ is the measured contact angle, θ_Y_ is the intrinsic Young’s contact angle, and *r* is the roughness ratio (ratio between the actual and projected solid surface areas). From the model, it can be deduced that the surface roughness is directly proportional to the wettability of the original surface i.e., hydrophilic surface becomes more hydrophilic and the hydrophobic surface becomes more hydrophobic on increasing the surface roughness. In our case, the growth of the CuO microtufts on the fractal surfaces leads to the superhydrophilic surfaces, which are directly in line with the Wenzel model. However, it is very difficult to calculate exact contact angles on the leaf skeleton surfaces because of the 3D architecture of microscale fractals and the uneven surface of the leaf skeleton at macroscale. Therefore, to show the wettability of the surfaces, the water spreading on the fractal surfaces was investigated using the droplet spreading experiments. The droplet spreading of 3 μL water droplets on the fractal surfaces having CuO microtufts can be seen in Video V1, Supporting Information. As soon as the water droplet touches the surface, the droplet instantaneously spreads and displays an extremely low contact angle. It can be seen that the water keeps on spreading across the fractal structures and eventually disappears after approximately 4 min. On the hydrophobic surfaces bearing CuO microtufts, the droplet stays in the spherical cap, which can be seen in Video V2, Supporting Information. This experiment was also conducted on the fractal surfaces having just Cu coating, which can be seen in Video V3, Supporting Information. In this case, the water droplet spreads on the surface very slowly and maintained a contact line over a period. This confirms that the CuO microtufts are responsible for the superhydrophilicity of the surface.

### Fog Harvesting Studies

The CuO microtufts, fractals of the leaf skeleton, and the interconnection of veins present two phases in the efficient collection of water. The microtuft structures help in the initial droplet capture and nucleation. The fractal-like structures aid in the droplet growth and swift movement of the water. Finally, the leaf vein-like structures aid in the efficient transport and collection of water.

**Figure 3:**
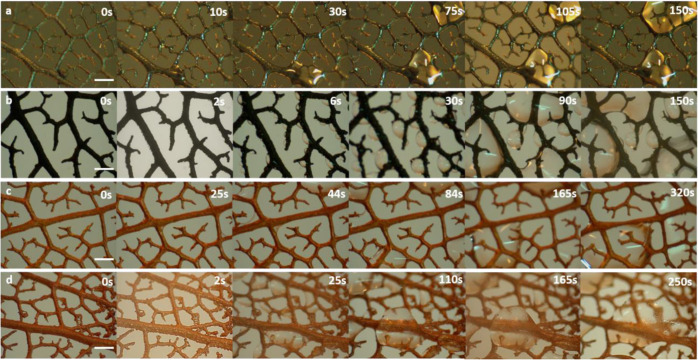
Snapshots of initial nucleation, droplet formation on fractal surface bearing (a). CuO microtufts, (b) CuO microtufts with hydrophobic coating, (c) bare Cu only, and (d) Cu only with hydrophobic coating. The scale bar in all cases is 200 μm.

The fog harvesting ability of the CuO microtufts embedded leaf skeleton along with the control samples was estimated in three independent cycles. The details of the experiment are given in the Materials and Methods sections. The leaf skeleton with just Cu coating was used as a control. Figure 3 shows the characteristics snapshots of the fractal structures during the initial nucleation cycle. In the case of the surfaces bearing the CuO microtufts, the surface is wetted instantaneously as soon as the first fog droplets hit the surface (Figure 3a). As the fog stream continues to reach the surface, the new fog droplets coalesce with the water already deposited over the surface and form a continuous film at least on the larger veins of the surface that are visible in the micrograph. This film remains constant even if more fog droplets are captured by the surface as there is a constant water movement on the surface, which can be seen in Video V4 (Supporting Information). In the case of the silanized CuO microtufts on the leaf skeleton, tiny droplets are immediately visible as soon as the fog stream touches the surface. In a typical micro/nanostructured hydrophobic surface, jumping off of the droplets is usually observed on the silanized surfaces.^46^ However, in this case, the nucleated droplets continue to grow due to the coalescence with the nearby droplets. It is evident from Figure 3b and Video V5 (Supporting Information) that the fractal-like geometry aids in the coalescence of the growing droplets and avoids shedding or jumping off of smaller droplets prematurely. After a few seconds, droplets with sizes ranging from tens of micrometers to a few millimeters are observed in this case. In the case of control hydrophilic Cu-coated skeleton, a film of water can be evidenced as seen in Figure 3c and Video V6 (Supporting Information). Unlike the hydrophilic surface with CuO microtufts, water remains on the surface and continues to grow further starting from the fractal curves. With time, the water takes the shape of a large droplet that remains pinned to the surface. In the case of silane-coated, Cu-coated fractal surface (Figure 3d and Video V7, Supporting Information), several small droplets could be observed, which eventually grow bigger and take a spherical shape.

**Figure 4:**
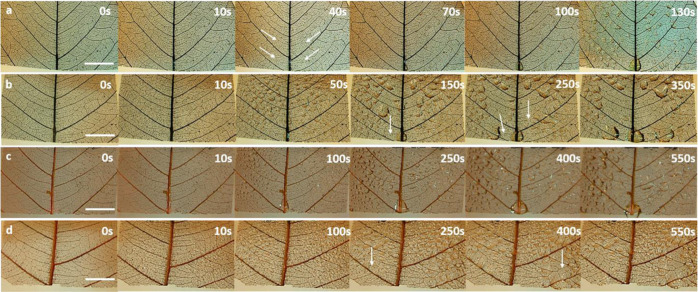
Snapshots showing water transport on the leaf veins bearing (a) CuO microtufts, (b) CuO microtufts with hydrophobic coating, (c) bare Cu only, and (d). Cu only with hydrophobic coating. The scale bar in all cases is 10 mm.

Figure 4 shows the characteristics snapshots of the CuO- and Cu-coated leaf skeleton surfaces during the transport of the captured water. In the case of the hydrophilic surfaces bearing CuO microtufts, the water travels across the leaf veins. The network of the fractals directs the continuous water film to the central vein where the water accumulates and can be collected as seen in Figure 4a. A similar water movement can be seen in the control Cu-coated hydrophilic surfaces (Figure 4b), where the water travels across subveins to the main central veins and gets accumulated at the center before being collected. However, the overall collection time was slow and bigger droplets can be seen across the surface of the control hydrophilic sample.

Figure 4c shows the water transport on the skeleton surfaces having silanized CuO microtufts. In a typical hydrophobic surface, perfectly spherical droplets are expected which either jump off the surface or roll off the surface quickly. However, in this case, the droplets initially appear spherical, but with time, they take random shapes as these continue to grow. This could be due to the fractal-like structure that does not allow droplets to jump and roll off quickly. These droplets coalesce with each other as expected in a meshlike surface. The droplets grow bigger and remain pinned to the surface for a prolonged period, which can be due to the instabilities in their Cassie states. Eventually, the droplets become bigger and slide through the surface with the aid of gravity. Unlike in the hydrophilic surfaces, the leaf veins do not tend to play a major role in the transport of the water. A similar trend was seen in the control silanized Cu-coated skeletons where the grown droplets slide down through the surface (Figure 4d). The time taken in this case is longer in comparison to the hydrophobic skeletons bearing CuO microtufts.

**Figure 5:**
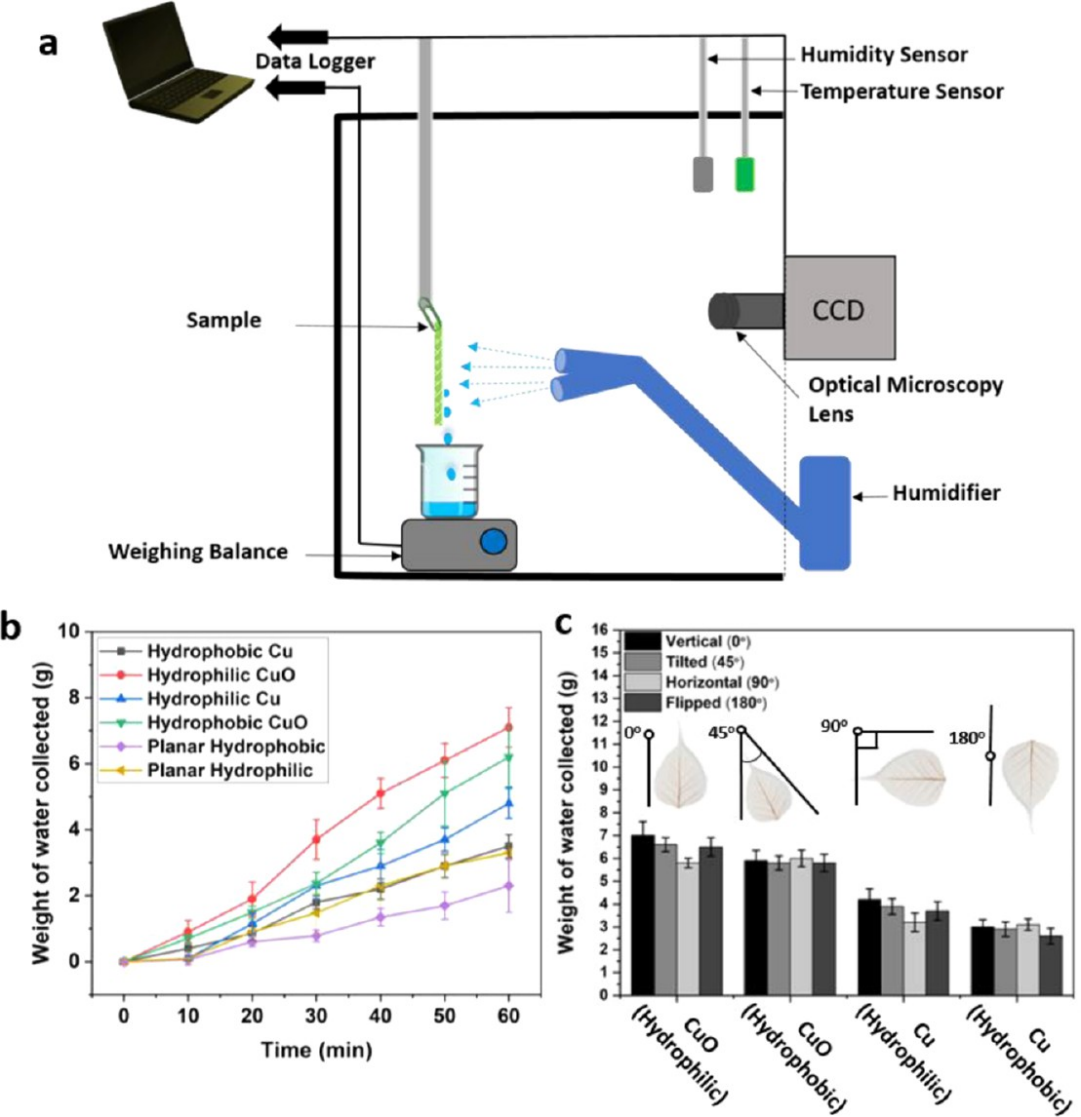
(a) Schematic of the water collection setup. (b) Fog harvesting dynamics as volume of water collected (g) in time, *t* (min), over skeleton surfaces of dimensions 40 mm × 40 mm. (c) Overall water collected over the period of 1 h when samples were tilted in the vertical (0°), tilted (45°), horizontal (90°), and flipped (180°) positions. Error bars show the standard deviations calculated from three independent measurements.

The water capture and removal efficiency of the different surfaces is directly related to the different forces acting on the droplet and the architecture of the surface. To quantify the water collection performance on the leaf skeletons bearing CuO microtufts and control surfaces, the surfaces were subjected to fog flow in a homemade setup as shown in Figure 5a. All of the samples were cut in a 40 mm × 40 mm dimension. The amount of water dripping from the leaf skeletons was quantified every 10 min, and the data are presented in Figure 5b. Throughout the 1 h of the fog collection experiments, the leaf skeletons bearing the CuO microtufts without and with hydrophobic coating collected 7.3 ± 0.6 and 6.5 ± 0.9 g of water, respectively. Normalizing this to the sample size, the overall efficiency in terms of water collected per unit area was calculated as 0.45 and 0.39 g cm^–2^ h^–1^ for the CuO microtuft leaf skeletons without and with hydrophobic coating, respectively. The hydrophilic skeleton surfaces bearing the CuO microtufts collected more water compared to the hydrophobic surfaces. The collection of water was also continuous throughout the water collection cycle in the case of hydrophilic skeletons with CuO microtufts having linear regression, *r*^2^ ≈ 0.99 as seen in Figure 5b. Control surfaces on the other hand collected a significantly lesser amount of water compared to the CuO microtufts bearing leaf skeletons. This is in contrast to the slow nucleation of water droplets at the surfaces that can be seen in Figures 3 and 4. The overall quantities of water collected were 4.8 ± 0.5 and 3.1 ± 0.4 g for skeletons with Cu coating and Cu-coated skeleton with hydrophobic coating, respectively. The water harvesting efficiency was 0.30 g cm^–2^ h^–1^ for the leaf skeletons with only the Cu coating, and 0.19 g cm^–2^ h^–1^ for the leaf skeletons with the Cu coating and the hydrophobic silane coating. In addition, hydrophilic and hydrophobic planar controls (CuO on glass substrate) having dimensions 40 mm × 40 mm were also tested for water collection. The overall water collection in an hour was 3.3 ± 0.4 and 2.3 ± 0.4 g for the hydrophilic and hydrophobic samples, respectively. The planar surfaces displayed normalized water harvesting efficiencies of 0.20 and 0.14 g cm^–2^ h^–1^ for hydrophilic and hydrophobic surfaces, respectively.

To quantitively check the role of the microtufts, hydrophobicity, and vein orientation in the water transport, samples were exposed to mist in vertical (0°), tilted (45°), horizontal (90°), and flipped (180°) positions, as shown in the inset of Figure 5c. The water was collected and quantified according to the setup shown in Figure 5a. Overall, the water collection was maximum with superhydrophilic surfaces with CuO microtufts and minimum with hydrophobic Cu-coated skeletons. To confirm if the effects of the CuO microtufts, hydrophobicity, or direction of the sample on the water collection efficiency were statistically significant, we conducted a three-way ANOVA test for the data (Supporting information Table S1). The microtufts (*P* < 0.001) and hydrophobicity (*P* < 0.001) have a statistically significant effect on the water collection efficiency. The orientation of the sample had a less significant effect but still had a *P*-value of 0.01. In the case of the superhydrophilic surfaces, we noted that the water collection was maximum when the samples were in the vertical position (most of the veins pointing downward). The horizontal position had the lowest yield, which could be correlated to the orientation of the veins. The water collection was on average ∼20% less when the surface was tilted horizontally than in the vertical position. In the case of skeleton surfaces having hydrophobic coatings, the orientation had a negligible influence on the water collection. This was also confirmed with two-way interaction between the direction of the leaf and hydrophobicity, which was found significant (*P* = 0.004). Hence, in hydrophobic leaf skeletons, only the presence of the CuO microtufts tends to play a role in the water collection efficiency rather than the vein orientation. However, it is noteworthy to mention that when the surfaces are designed in contrasting wettability, the vein orientation may play a major role in water transport.

**Figure 6:**
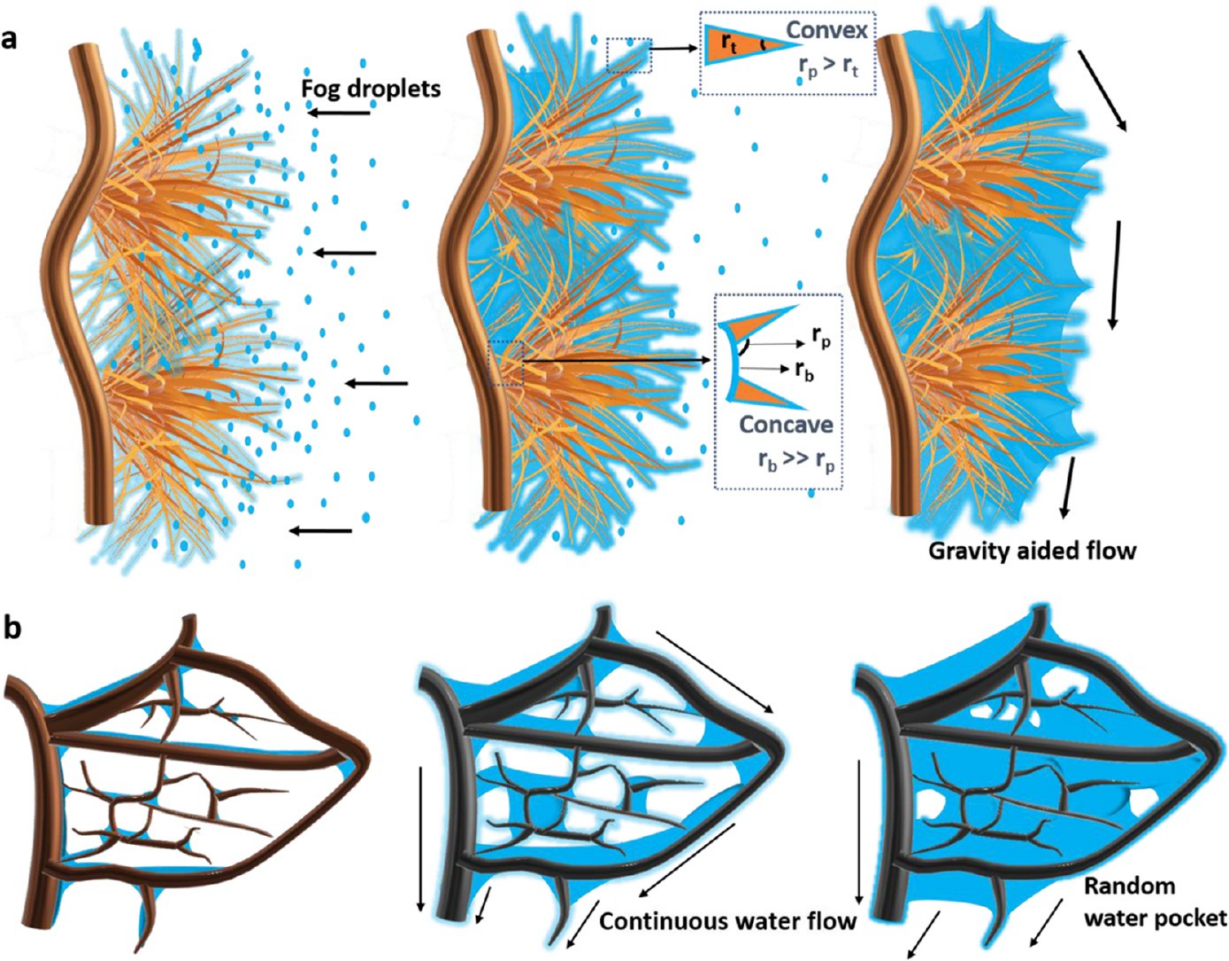
(a) Mechanism of initial nucleation and microtufts. (b) Schematic of water growth and movement in CuO microtufts bearing fractal surfaces.

In this study, the unique microstructures, fractals, and skeleton veins play a major role in the overall water harvesting efficiency. The water harvesting mechanisms, in this case, can be divided into nucleation, growth, and transport. The initial nucleation and growth of water in CuO microtufts can be explained by the schematics in Figure 6. The morphology and the wettability of the surfaces have a direct impact on the water harvesting ability. Under normal circumstances on the surfaces devoid of any micro/nanostructures, it starts with the tiny droplets condensation on the surface, which gradually becomes larger with further condensation as reported in ref (7). This is followed by the further coalesce of the deposited droplets of random size and shape with the neighboring drops, which eventually leads to the formation of the liquid film over the surface (Figure 3c and Video V6). The flow of these water films with the aid of gravity was observed to be very slow, which is leads to the water clogging between the fractal surfaces and responsible for the comparatively low water harvesting on these surfaces. On the leaf skeletons bearing CuO microtuft structures, a thin film of water was immediately observed on the surface as soon as the surface was exposed to the fog flow. Microtuft-like shape is composed of long conical thread-like fragments originating from a common base. Hence, the water collection on the individual fragment can be defined via pressure gradients on the conical structures. For all microconical fragments where the radius of the cones increases from tip to base, the pressure gradient due to the surface tension on the film of water is^47,48^
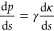
5

Here, *p* is the liquid pressure (water), *s* is the position of the fibers, κ is the curvature, and γ is the surface tension. Due to this pressure gradient, water migrates from the convex tips to the concave parts between the fibers of the microtufts. According to eq 5, liquid pressure decreases along the direction of the increasing radius on microfibers. The liquid pressure also decreases along the direction of a declining radius between the microfibers. Therefore, the water condensed on the tip of the microfiber will be pulled toward the periphery (lower radius of the microfiber). In the same way, the water deposited around the periphery of the microfiber will be drawn toward the bottom surface. The radius of the microfiber tip *r*_t_ is smaller compared to the radius of microfiber periphery *r*_p_ (*r*_p_ > *r*_t_). Also, the radius of the base *r*_b_ is much larger compared to the periphery (*r*_b_ > *r*_p_, see Figure 6a). This means that the water gets deposited as a continuous film. This film will be pulled toward the region near the periphery of the microfiber by the influence of the pressure gradient. Minimal overflooding was observed, which could be due to the assembly of the long microfibers in the microtuft-like orientation and the proximity of numerous microtufts. The decrease in the overflooding on the hydrophilic surfaces leads to an increase in condensation coefficient and a better water collection efficiency.^48,49^

The hydrophilic skeleton surfaces bearing CuO microtufts displayed a unidirectional water movement throughout the veins. The movement was swift and continuous as seen in Figure 4a. The venation pattern of the leaf skeleton is such that the smaller veins keep on increasing the order and eventually merge with the central vein. There is a progressive increase in the diameter of the veins from the primary to the secondary level. So, when the water film is formed on the superhydrophilic surface during the initial phase, it leads to the Young–Laplace pressure difference between the veins.^17^ This will lead to a continuous water movement from smaller veins to the bigger veins. The shape of the veins provides the continuous curvature gradient, which furthermore aids in the smooth movement of the water at the turns.^39^ This arrangement ensures that there is no backflow of the merged water and the unidirectional transport of the water is maintained along the veins. However, there were places within the fractal structures with uneven bumps or uneven thickness. At those places, the water gets accumulated, leading to small droplets/water pocket-like appearance. However, these water accumulations did not affect the overall water collection and the overall movement was swift as evidenced in Figure 4a.

**Figure 7:**
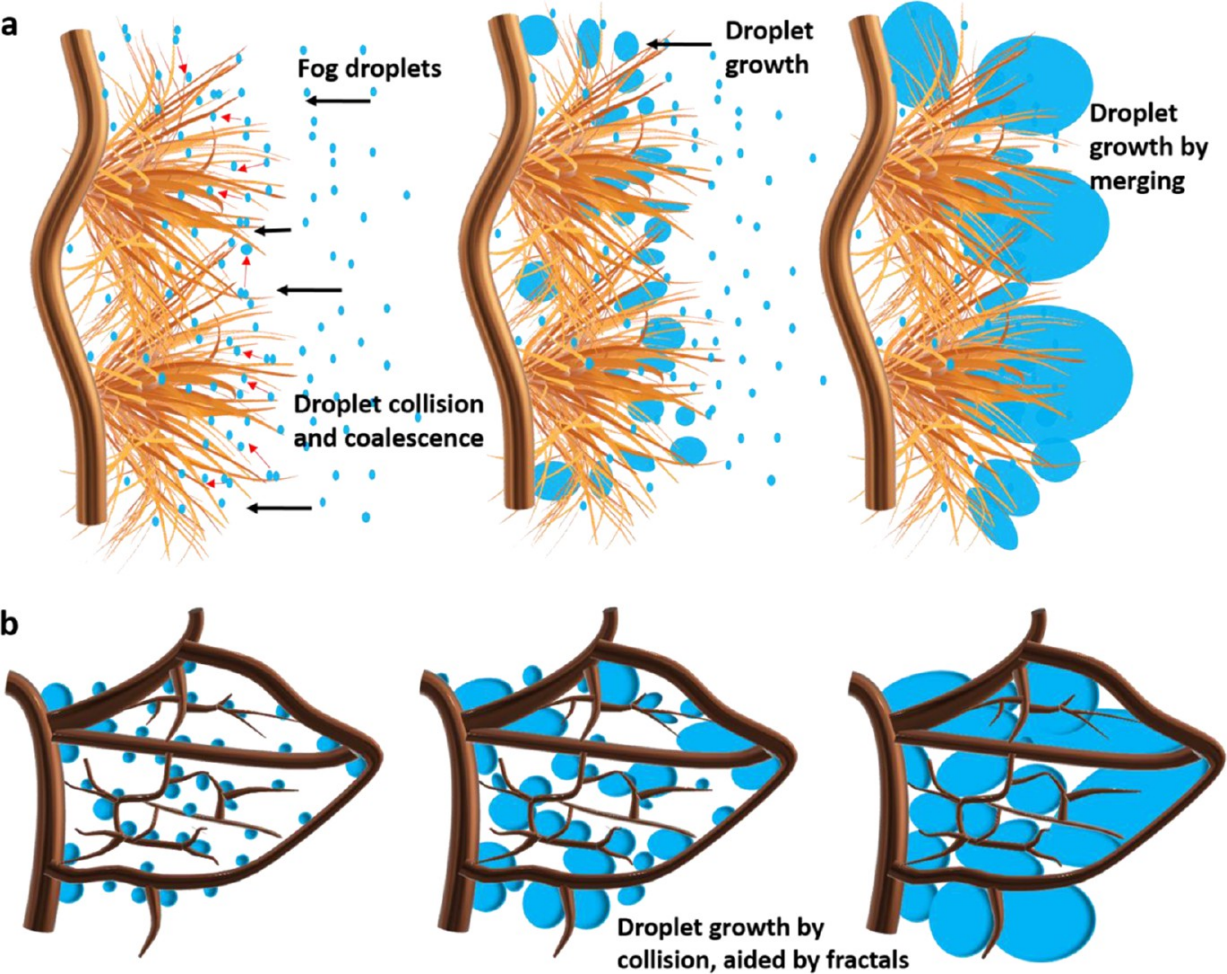
(a) Schematic of initial droplet nucleation and growth on hydrophobic CuO microtufts bearing fractal surfaces. (b) Droplet growth in the case of hydrophobic CuO microtufts bearing fractals.

On the hydrophobic leaf skeletons bearing CuO microtufts, a different type of water collection was observed. The CuO microtufts possess a higher surface area and have more nucleation sites for the initial fog droplet condensation (see Figure 7a). The nucleated and coalesced droplets either jump off or roll down the low-energy microfiber surface. Due to the high surface area and arrangement of the microfibers in the microtuft-like structure, the probability of the droplets merging with other droplets increases. This type of assembly also ensures that there is minimal loss of water droplets from the surface due to the fog flow and maximum droplets are trapped. The silane coating may also lead to the jumping off of the droplets, and the droplets that jump off the microfiber surfaces get trapped by neighboring microfibers. These coalesced droplets continue to grow quickly, as evidenced in Figure 3b. The fractal structures display the high surface area and maximum surface coverage across the leaf skeleton.^50^ This ensures that these droplets growing at multiple sites further coalesce/collide with each other to form the bigger droplets (Figures 3b and 7b). Ideally, in the superhydrophobic surfaces, it has been seen that the droplets that fall off the surface are tiny and the shedding is quick.^7^ However, in this case, these tiny droplets gradually became bigger to eventually merge with the other droplets. When the droplet attained a critical size, instead of rolling off, it slides off the surface. The critical size of the droplet pinned to a surface under the influence of gravity can be estimated using the following equation^51^
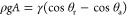
6Here, ρ is the density of the droplet, γ is the surface tension, *A* is the cross-sectional area of the drop, θ_r_ and θ_a_ represent the receding and advancing contact angles, respectively, and *g* denotes the gravitational force. The surface displayed large droplets during fog harvesting experiments because of its high contact angle hysteresis, which is evident in Figure 4b. In the case of hydrophobic skeleton surfaces without any microtufts, comparatively fewer droplets got nucleated initially due to the surface being a barrier to the fog flow. The initial deposition process was comparatively slow, as seen in Video V7, Supporting Information. The captured droplets grew in the same way as the hydrophobic skeletons bearing CuO microtufts, aided by the fractal structures. When the droplet attains a critical size, it slides off the surface. The droplet sliding and the shedding process were much slower in this case. This proves that the presence of the CuO microstructures facilitates the initial fog droplet capture and deposition onto the surfaces.

To check how mechanical damage affects the fog collection performance, we conducted the sandpaper wear test. First, the sample was placed on sandpaper (grade: P120) to make a contact and a weight of 400 g was placed on the surface. The sample was pushed ∼5 cm in one direction and then rotated 180° and moved ∼5 cm in the opposite direction. Each 10 cm was noted as a cycle, for a total of 25 cycles. The camera and SEM images of the worn sample are shown in Figure S7. The abrasion is evident from the camera images. However, on close inspection of the SEM images, it can be concluded that the abrasion occurs only at the top of the surfaces. There are significant areas where the CuO microtufts can be seen. This is due to the three-dimensional surface architecture of the fractals that provide shielding against the abrasion under normal circumstances. There was no significant difference in the overall fog collection efficiency of the abrated surfaces compared to the original surfaces, which can be seen in Figure S8.

Similarly, to check the long-term stability of the fabricated surfaces, fog collection experiments on ∼100-day-old samples were conducted. The SEM images of the ∼100-day-old samples, which were used in the fog collection studies, are shown in Figure S9. It can be seen that the microtufts got converted to the random microstructures after 100 days and three cycles of the fog harvesting tests. This might be due to the further oxidation of the surfaces over time in the presence of airborne impurities. The fog collection on the samples (∼100 days old) is presented in Figure S10. There was a notable change in overall water collection efficiency in the case of hydrophilic surfaces. In general, superhydrophilic CuO micro/nanostructures tend to become hydrophobic over time due to the adsorption of the airborne carbon species.^52^ However, the fractal structures and the already hydrophobic surfaces bearing CuO microtufts maintained a comparable performance over time. Taking all of these results together with the results shown in Figure 5b, the results suggest that while the hydrophobic coating reduces the initial fog collection efficiency, it may make the CuO microtufts more stable over long periods.

The above observations, analysis of water collection on leaf skeletons with CuO microtufts, and comparison with the literature reports suggest that multilevel water-collecting networks display hierarchical structures that play various roles in water collection. Micro/nanostructures, such as the CuO microtufts in this work, aid in the initial capture of the fog droplets from the fog stream.^7,48^ In the case of superhydrophilic surfaces, these microstructures expand the area of the surface that encounters the fog/mist stream, which enhances the capture of the fog droplets. The wettability gradient arising from the microtufts drives the water film along the skeleton surface. In addition, the fractal structures aid in efficient water movement. Finally, the veins play a major role in the efficient collection of the captured water by enhancing/facilitating unidirectional transport. In hydrophobic surfaces also, the CuO microtufts and fractal structures aid in the quick nucleation and droplet growth. The meshlike shape eventually leads to further droplet growth until the droplet slides off the surface.

The quantitative water collection results indicate that if subjected to a large scale, the surfaces can harvest a decent amount of water for human consumption. Comparing with the reported copper and copper oxide-based fog harvesting surfaces, CuO microtuft-based surfaces showed a comparable fog harvesting efficiency (see Table S2, Supporting Information). In addition, the leaf surfaces are in abundance and the integration of CuO microtufts onto them is facile, which makes their mass production possible. It is also noteworthy to mention that the leaf skeletons of *F. religiosa* used in this study are not the only natural surfaces that can be used for this application. Many leaf skeletons display different fractal architectures at the micro and macroscales, and our deposition technique should apply to any kind of leaf skeletons.^53,54^ Some unique fractal architectures may aid in even better water collections and some fractal architecture may hinder the droplet formation and efficient water collection. However, the technique is limited to leaves that can be skeletonized: leaves from many plant species are fragile and tend to break when they are skeletonized, so it is difficult to produce the freestanding fractal structures from them.

## Conclusions

In this study, we have exploited a new concept of producing microtufts onto the fractal-like leaf skeletons to demonstrate rapid nucleation, droplet growth, and unidirectional transport. First, copper oxide microtufts were fabricated onto the *F. religiosa* leaf skeletons via electroplating and chemical oxidation techniques. The fabricated surfaces with microtufts displayed high wettability and excellent water capture. A silane coating was applied to the surface to make the surface hydrophobic. Fog harvesting experiments were conducted on hydrophilic and hydrophobic surfaces. The overall water collection efficiencies were determined, and the role of the microtufts, fractal structures, and the orientation leaf veins was investigated. About 52 and 85% increases in fog harvesting efficiency were achieved with superhydrophilic and hydrophobic leaf skeleton surfaces bearing CuO microtufts, respectively, compared to the planar control surfaces. The combination of microstructures with controlling roughness with the unique fractal-like architecture of the leaf skeleton enables the most effective mechanism in water collection in both the superhydrophilic and -hydrophobic surfaces. On surfaces without silane coating, the CuO microtufts display excellent wettability and unidirectional water transfer aided by fractals and veins. In surfaces bearing the silane coating, the CuO microtufts aid in rapid nucleation and growth of the water droplet, which aids in efficient water harvesting. Future studies will include the study on the different leaf skeleton-based designs and their biomimetic replication to achieve even better water harvesting efficiencies.

## Supporting Information Available

SEM images of CuO nanograss on glass substrates; EDS mapping of leaf skeletons having different coatings; SEM images of abrated surfaces and 100-day-old surfaces; fog collection results on the abrated surfaces and 100-day-old samples; analysis of variance; and comparison of fog collection performance (PDF)
Wettability video; droplets on the fractal surfaces having CuO microtufts (MP4)
droplet stays in the spherical cap (MP4)
fractal surfaces having just Cu coating (MP4)
more fog droplets are captured by the surface as there is a constant water movement on the surface (MP4)
nucleated droplets continue to grow due to the coalescence with the nearby droplets (MP4)
control hydrophilic Cu-coated skeleton, a film of water (MP4)
silane-coated, Cu-coated fractal surface, several small droplets could be observed, which eventually grow bigger and take a spherical shape (MP4)
