# Supplementary material for: Copper Oxide Microtufts on Natural Fractals for Efficient Water Harvesting
Source: Langmuir. 2021 Mar 11;37(11):3370–81. doi: 10.1021/acs.langmuir.0c03497 (PMC8043393; doi:10.1021/acs.langmuir.0c03497)
Supplement: Supplementary file 1 — la0c03497_si_001.pdf [file la0c03497_si_001.pdf]

Copper Oxide Micro-Tufts on Natural Fractals for Efficient Water Harvesting

Vipul Sharma[1]*, Harri Ali-Löytty[2], Anastasia Koivikko[1], Kyriacos Yiannacou[1], Kimmo Lahtonen[3] and Veikko Sariola[1]*

[1]Faculty of Medicine and Health Technology, Tampere University, Finland

Korkeakoulunkatu 3, 33720 Tampere, Finland

[2]Surface Science Group, Photonics Laboratory, Tampere University, P.O. Box 692, FI-33014 Tampere University, Finland

[3]Faculty of Engineering and Natural Sciences, Tampere University,

P.O. Box 692, 33014 Tampere, Finland

*Email: vipul.sharma@tuni.fi, veikko.sariola@tuni.fi

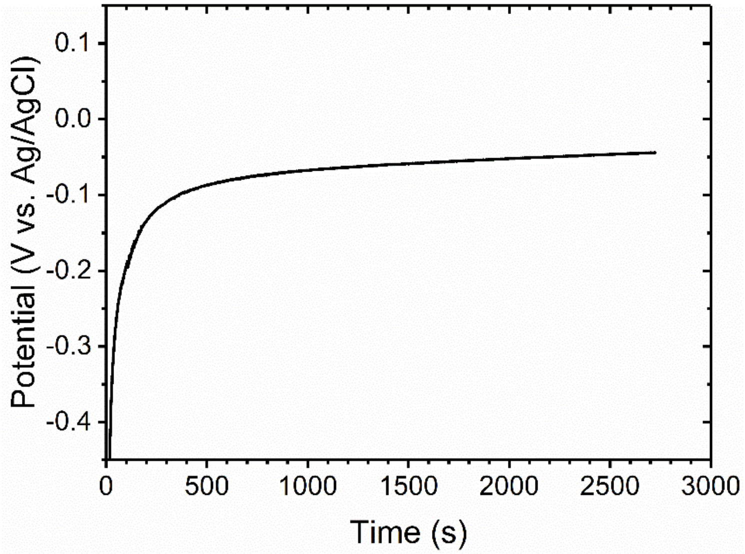

Figure S1. Potential as a function of time during galvanostatic electrodeposition of Cu on Au coated leaf skeleton at –10 mA/cm^2.

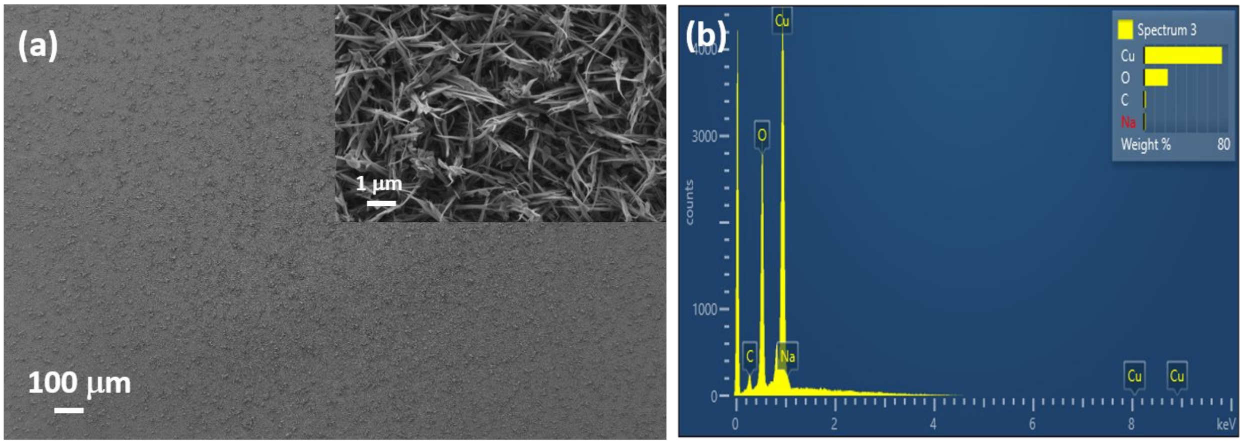

Figure S2. (a) FESEM image of the CuO grown on the glass substrate (planer surface). (b) Shows the corresponding EDS spectra.

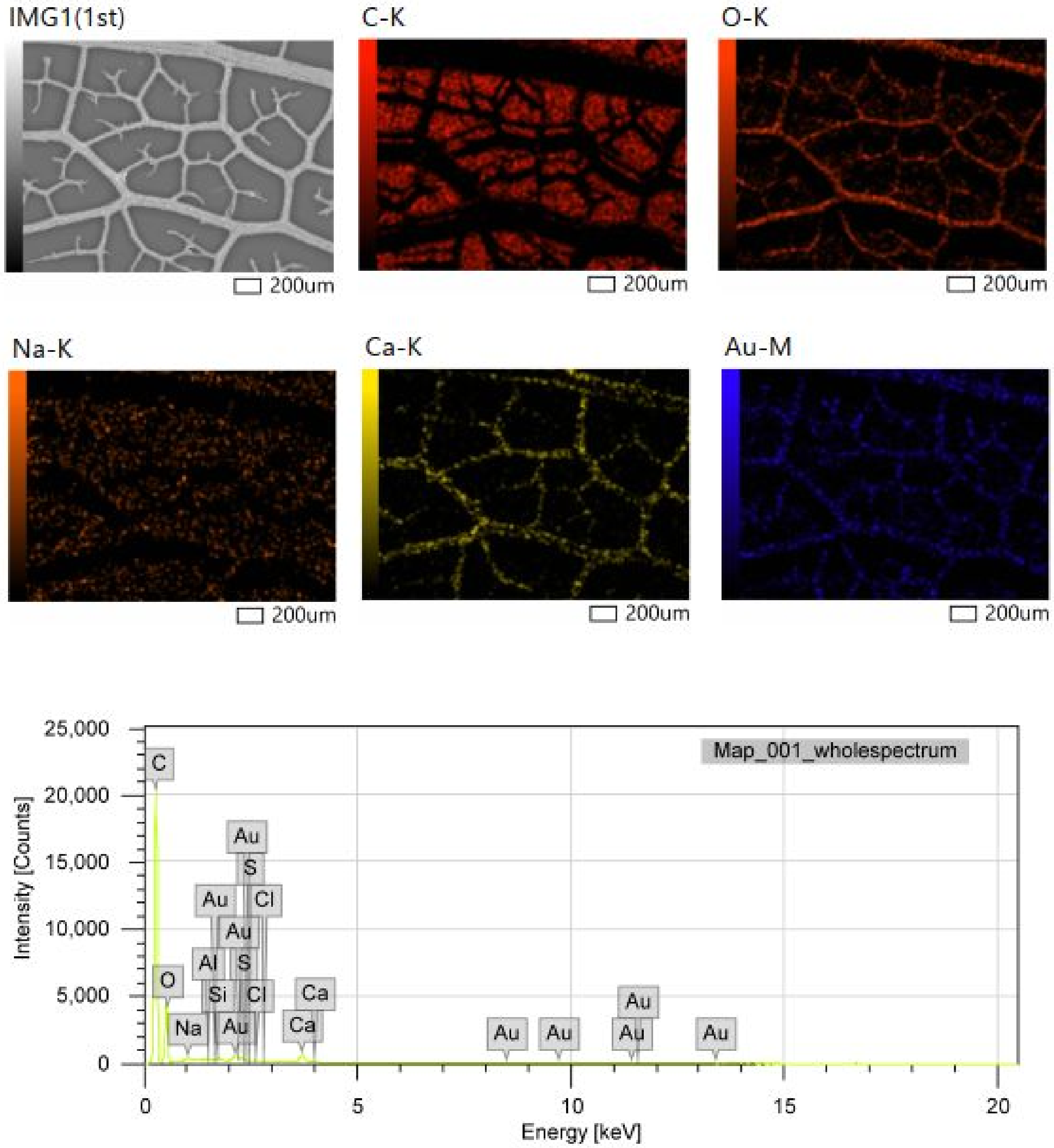

Figure S3. EDS mapping and corresponding spectra of the leaf skeleton after Au deposition.

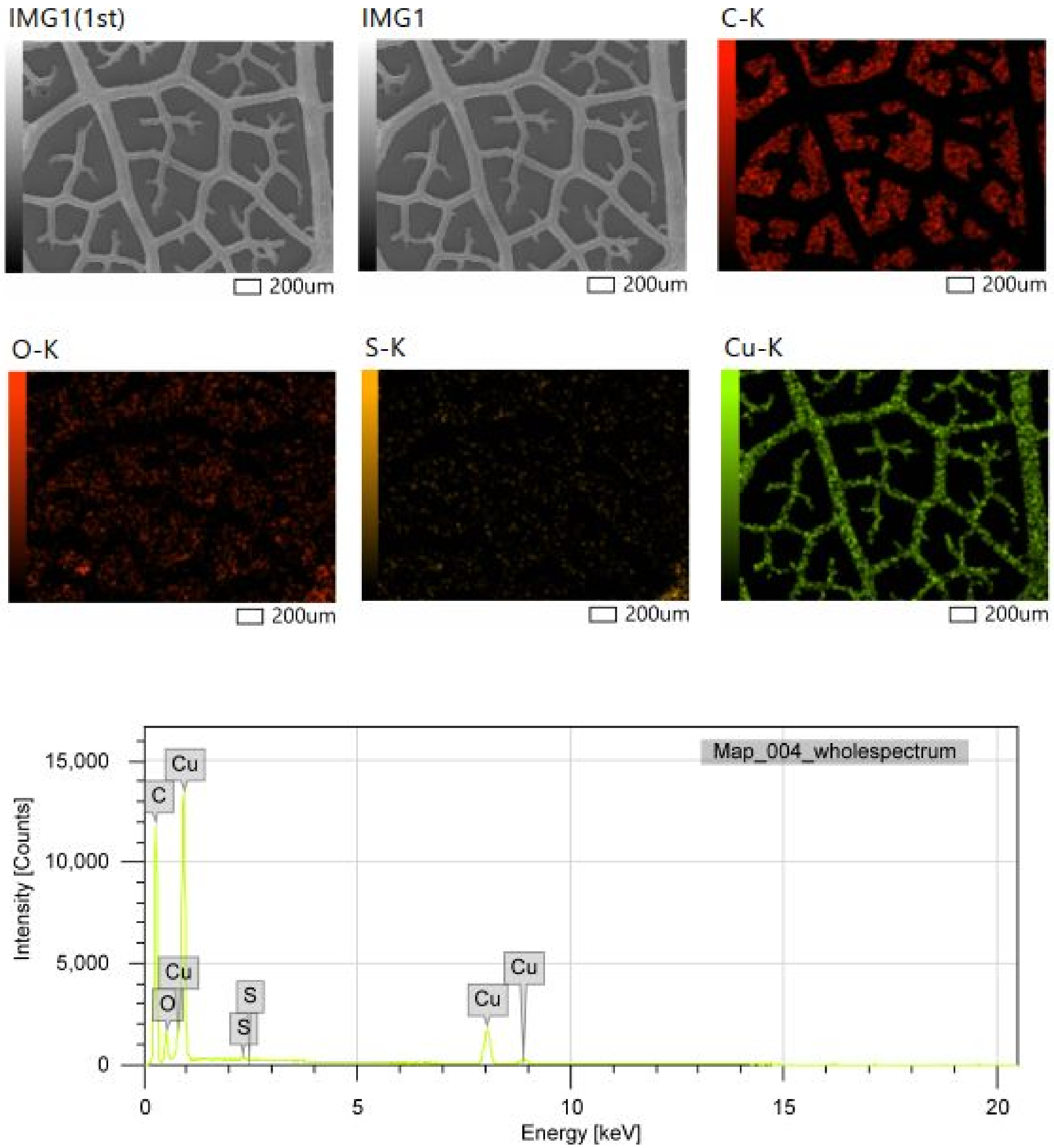

Figure S4. EDS mapping and corresponding spectra of the leaf skeleton after Cu deposition.

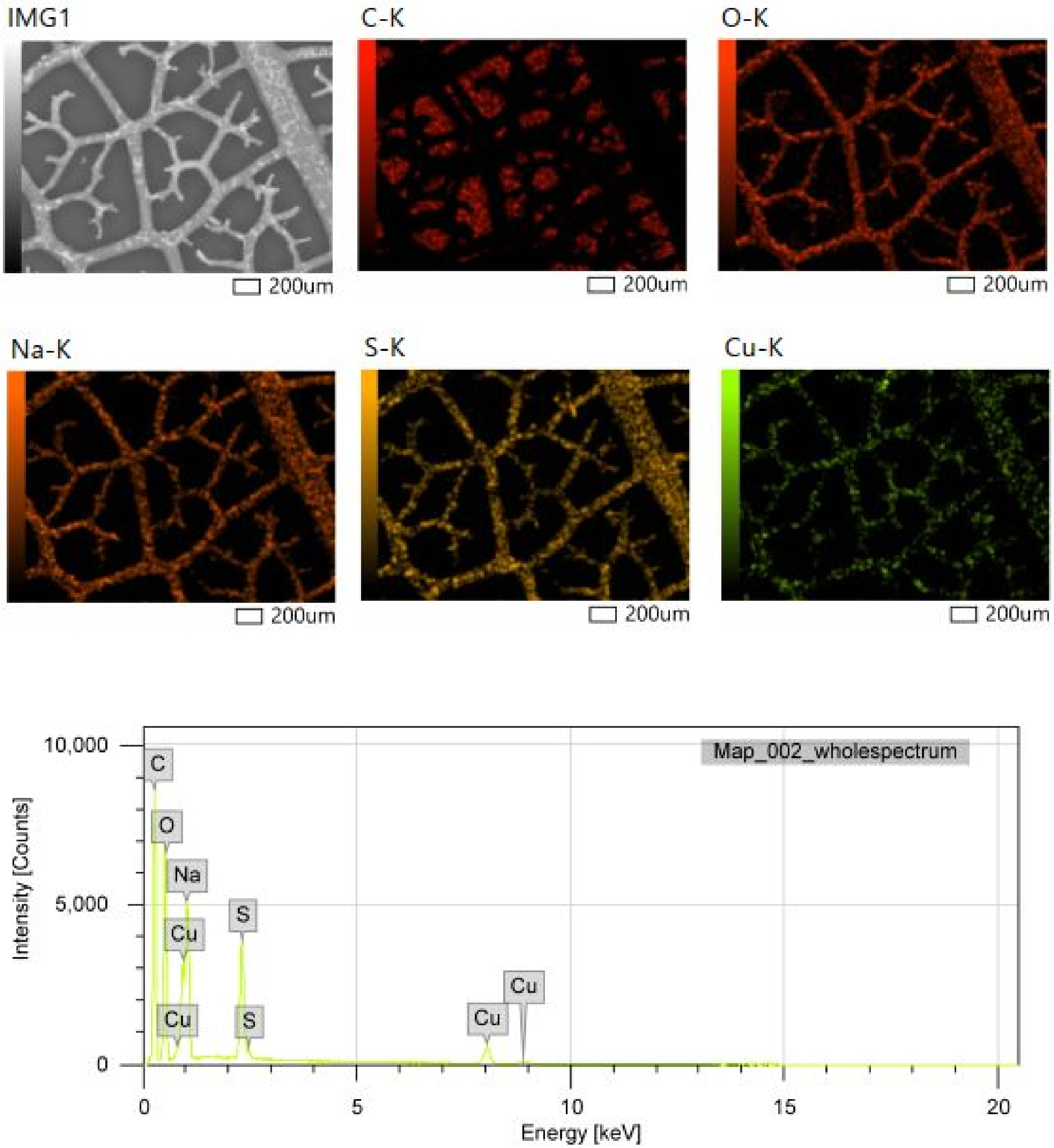

Figure S5. EDS mapping and corresponding spectra of the leaf skeleton after CuO microtuft fabrication on the surface via oxidation.

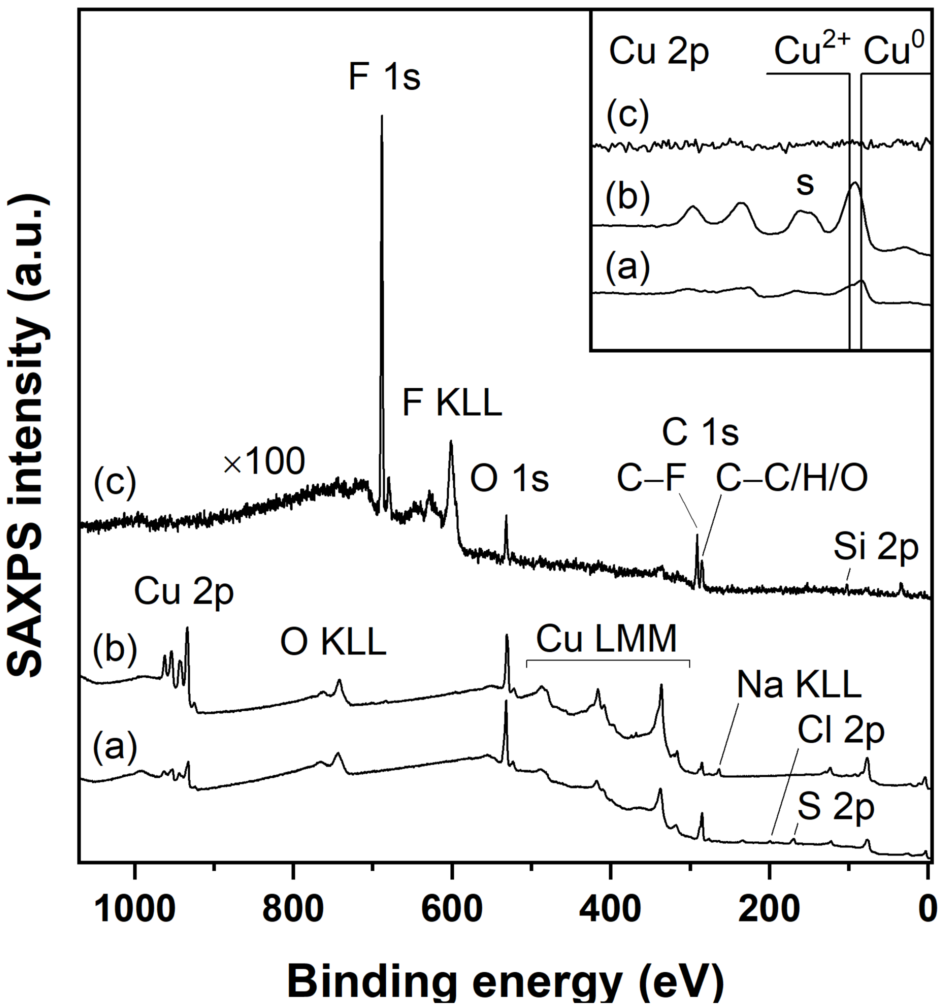

Figure S6. SAXPS survey spectra of **a.** Cu, **b.** CuO, and **c.** CuO silane (intensity × 100). The inset shows zoom-in of the Cu 2p region where "s"=Cu^{2+} shakeup. Note the low intensity and high noise level in **c.** Low X-ray flux was used to scan the spectrum because of X-ray induced outgassing (C–F bond breaking) of the sample.

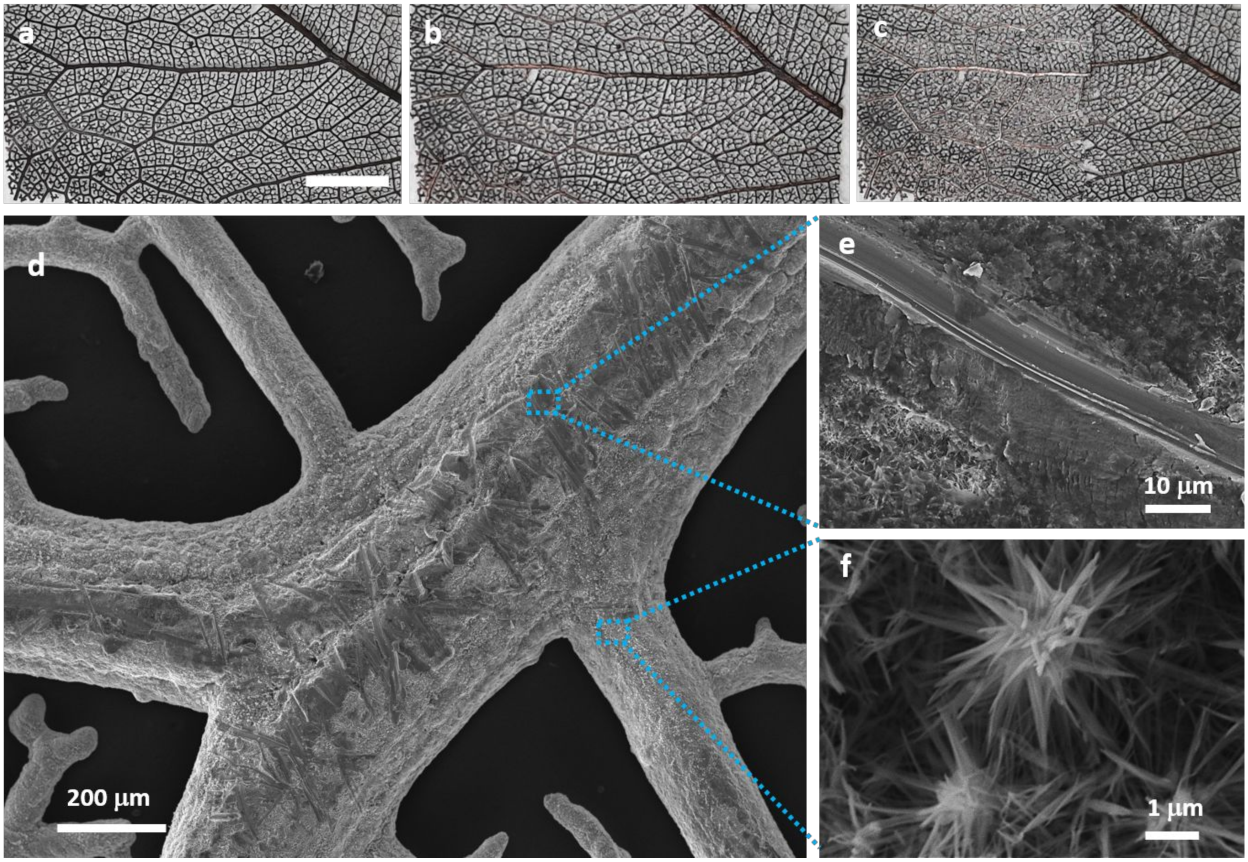

Figure S7. Camera images of the surfaces, **a.** before scoring with sandpaper, **b.** after mild abrasion (25 cycles) and **c.** heavy abrasion. The scalebar in camera images is 5 mm. **d.** shows the SEM image of the abraded sample (25 cycles). **e.** and **f.** show the areas with the abrasion signs and the shielded areas with the microtufts, respectively.

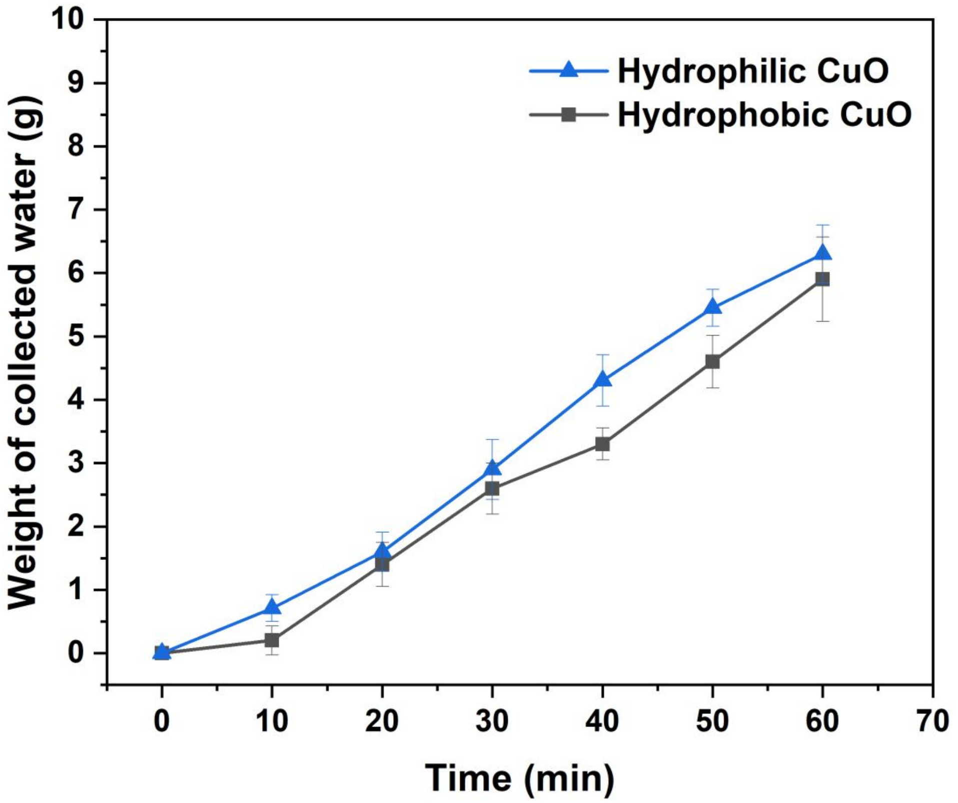

Figure S8. Fog harvesting dynamics as the volume of water collected (g) in time, t (min), over abraded skeleton surfaces (scored with sandpaper, 25 cycles) having dimensions 40 × 40 mm. Error bars show the standard deviations calculated from three independent measurements.

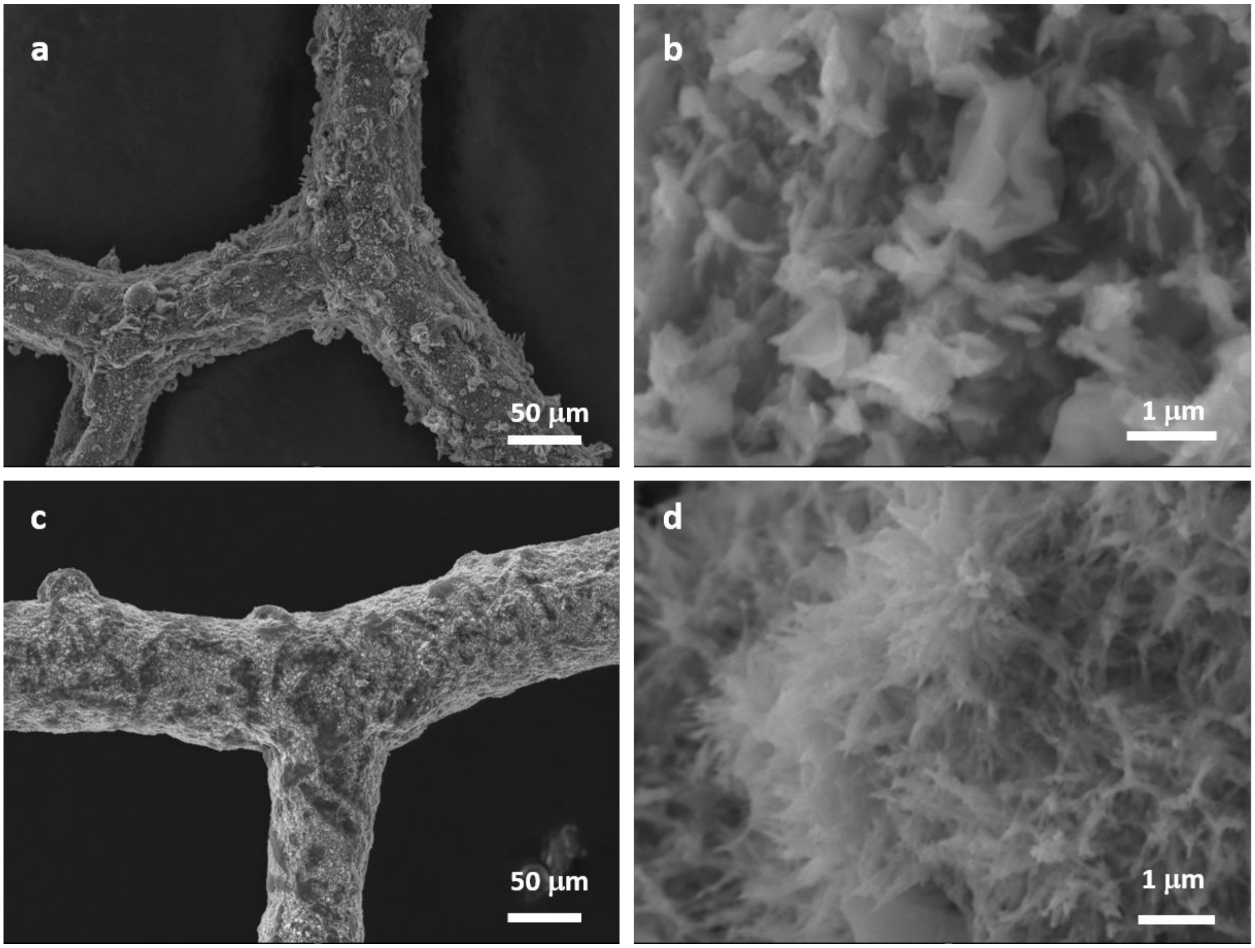

Figure S9. SEM Images of the 100 days old leaf skeleton bearing (a—b), CuO microtufts without silane coating, and (c—d) CuO microtufts with silane coating.

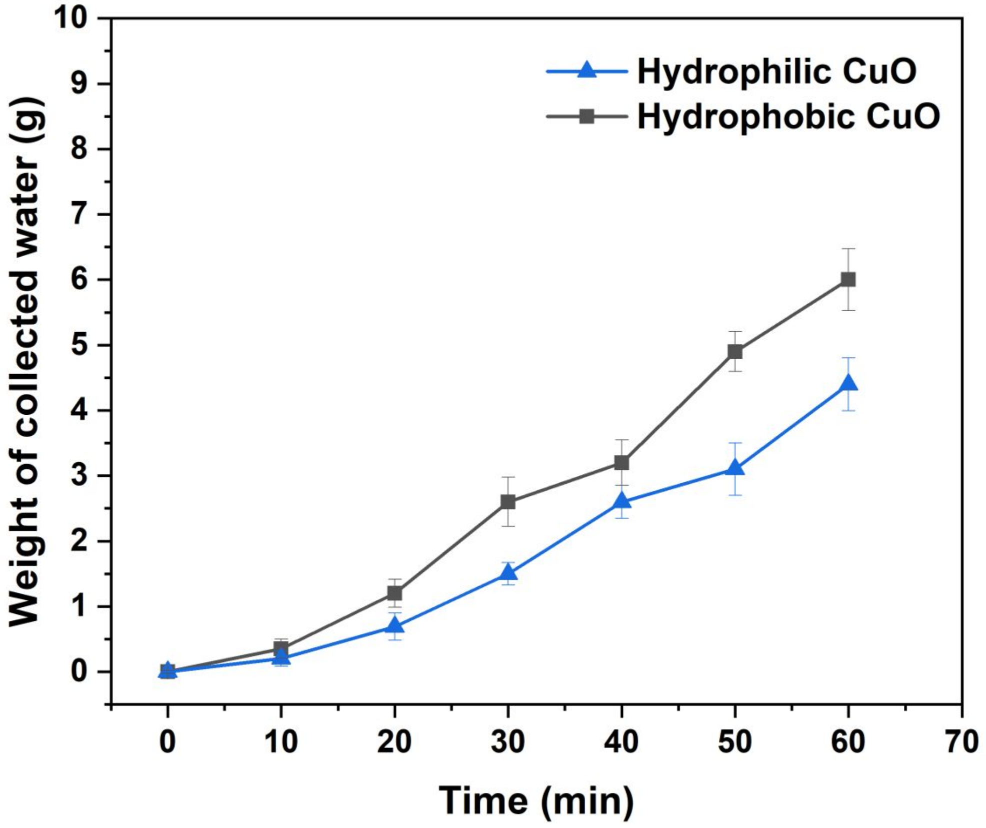

Figure S10. Fog harvesting dynamics as volume of water collected (g) in time, t (min), over 100 days old skeleton surfaces having dimensions 40 × 40 mm. Error bars show the standard deviations calculated from three independent measurements.

Table S1. Analysis of variance.

Source	Sum Sq.	d.f.	Mean Sq.	F	Prob>F
**Direction**	0.5652	3	0.1884	29.9	0.0098
**Hydrophobicity**	2.1219	1	2.1219	336.71	0.0004
**CuO/ Cu**	31.8472	1	31.8472	5053.63	0
**Direction (Hydrophobic)**	0.982	3	0.3273	51.94	0.0044
**Direction (CuO/Cu)**	0.565	3	0.0188	2.99	0.1962
**Hydrophobicity (CuO/Cu)**	0.747	1	0.747	11.86	0.0411

**Error**	0.0189	3	0.0063		
**Total**	35.6664	15			

Table S2. Comparison of fog collection performance of different Cu-based surfaces reported in the literature.

**S. No.**	**Fabrication Method**	**Fog flow rate (cm s^{-1})**	**Water collection rate (g cm^{-2} h^{-1})**	**Ref.**
1	Alternative thiol modification of TiO_2 and Cu	10	~1.309	1
2	Photocatalysis of CuO on a TiO_2 coated copper mesh	50	~0.571	2
3	Chemical oxidation of scorched Cu plate	5-15	~0.5	3
4	Chemically modified 1D Cu wires (~50 µm)	60	∼11.6	4
5	Electrochemical deposition and ammonia corrosion of Cu	~25	~0.707	5
6	Ammonia corrosion of Cu/ liquidus modification	~50	∼1.3	6
7	Electrochemical corrosion	240	~0.618	7
8	Ammonia corrosion / particle deposition	50-70	0.430	8
9	Alkaline oxidation/modification	N/A	1.317	9
10	Chemically modified Cu	25	~0.211	10

11	Electrodeposited CuO microtufts on leaf skeletons	5—15	0.45	This work

References:

(1) Zhu, H.; Guo, Z. Hybrid Engineered Materials with High Water-Collecting Efficiency Inspired by Namib Desert Beetles. *Chem. Commun*. **2016**, 52 (41), 6809–6812.

(2) Gou, X.; Guo, Z. Hybrid Hydrophilic−Hydrophobic CuO@TiO 2-Coated Copper Mesh for Efficient Water Harvesting. *Langmuir* **2019**, 36, 1, 64–73.

(3) Sharma, V.; Yiannacou, K.; Karjalainen, M.; Lahtonen, K.; Valden, M.; Sariola, V. Large-Scale Efficient Water Harvesting Using Bioinspired Micro-Patterned Copper Oxide Nanoneedle Surfaces and Guided Droplet Transport. *Nanoscale Adv.* **2019**, 1, 4025–4040.

(4) Zhong, L.; Zhang, R.; Li, J.; Guo, Z.; Zeng, H. Efficient Fog Harvesting Based on 1D Copper Wire Inspired by the Plant Pitaya. *Langmuir* **2018**, 34 (50), 15259–15267.

(5) Zhou, H.; Jing, X.; Guo, Z. Optimal Design of a Fog Collector: Unidirectional Water Transport on a System Integrated by Conical Copper Needles with Gradient Wettability and Hydrophilic Slippery Rough Surfaces. *Langmuir* **2020**. 36, 6801−6810.

(6) Zhong, L.; Feng, J.; Guo, Z. An Alternating Nanoscale (Hydrophilic-Hydrophobic)/Hydrophilic Janus Cooperative Copper Mesh Fabricated by a Simple Liquidus Modification for Efficient Fog Harvesting. *J. Mater. Chem. A* **2019**, 7 (14), 8405–8413.

(7) Xu, T.; Lin, Y.; Zhang, M.; Shi, W.; Zheng, Y. High-Efficiency Fog Collector: Water Unidirectional Transport on Heterogeneous Rough Conical Wires. *ACS Nano* **2016**, 10 (12), 10681–10688.

(8) Zhong, L.; Zhu, H.; Wu, Y.; Guo, Z. Understanding How Surface Chemistry and Topography Enhance Fog Harvesting Based on the Superwetting Surface with Patterned Hemispherical Bulges. *J. Colloid Interface Sci.* **2018**, 525, 234–242.

(9) Zhu, H.; Yang, F.; Li, J.; Guo, Z. High-Efficiency Water Collection on Biomimetic Material with Superwettable Patterns. *Chem. Commun*. **2016**, 52 (84), 12415–12417.

(10) Zhou, H.; Jing, X.; Guo, Z. Excellent Fog Droplets Collector via an Extremely Stable Hybrid Hydrophobic-Hydrophilic Surface and Janus Copper Foam Integrative System with Hierarchical Micro/Nanostructures*.* *J. Colloid Interface Sci*. **2020**, 561, 730–740.
